# Ligature of the External Iliac Artery for Femoral Aneurism

**Published:** 1842-10

**Authors:** Wm. Power

**Affiliations:** One of the Attending Physicians to the Baltimore Alms-house Hospital


*Ligature of the External Iliac Artery for Femoral Aneurism.* By Wm. Power, M. D., one of the Attending Physicians to the Baltimore Alms-house Hospital.—Christopher Daly, a native of Baltimore County, æt. 61, entered the surgical ward on the 1st of July; a man of stout frame, very youthful appearance for one of his years, and enjoying excellent health with the exception of the aneurismal disease for which he came to the hospital. For the last fourteen years he has enjoyed good health, uninterrupted by an attack of any sort unfitting him for his daily labor. About fifteen months ago, according to his best recollection, his attention was first accidentally called to the aneurismal tumor whilst digging in a ditch, and standing in water, though with his feet well protected. Placing his fingers upon it, he says he "distinctly felt a pulse, but thought it was in a very strange place."—As it gave him no pain or inconvenience he thought it was nothing serious and continued to work on as usual. About a month had elapsed from this time, when he was suddenly seized with violent cramps in the diseased limb, and finding exertion painful, he stopped his usual employment, but assisted during the following harvest in the lighter kind of field labor. The limb was slightly swollen below the tumor when he first discovered it; but after the attacks of cramp, which were from time to time repeated, the swelling increased, together with a sense of numbness diffusing itself over the whole leg. The tumor has steadily continued to increase, together with the œdema of the leg. He has, for the last eleven months, kept quiet and strictly avoided all exertion, and has undergone no treatment until he came under our care. The aneurismal tumor was situated in the right groin immediately below Poupart's ligament, which was forced a little upwards in an arched form. It was of an irregularly rounded form, about three and a half inches in its perpendicular, and three in its transverse diameter—pointing a little upwards and inwards, where the pulsation was more distinctly seen and felt than elsewhere. Its pulsation was evident to the eye—communicated a distinct thrill to the hand when laid upon it, a *bruit de rape* to the stethoscope, and the pulsations were eccentric. Compression upon the iliac artery, on the psoæ muscles, arrested all pulsation in the tumor. The external iliac for an inch above the tumor felt larger than the artery of the opposite side, and both here and in the continuation of the femoral below the tumor, gave a distinct file-sound to the stethoscope. The lymphatic glands of the groin were swollen and hard—the leg was one-third larger than that of the opposite side—purplish in hue, one degree higher in temperature, affected frequently with cramps, and pain with a sense of numbness over the knee and instep.

Tongue clean, appetite good, bowels regular, skin pleasant, pulse 80—natural, sounds of the heart clear and distinct, chest every where resonant, respiratory murmur vesicular, no ossification of the arteries at the wrist. The rest and quiet of the hospital reduced in a degree the œdema of the leg, and caused the tumor to appear more prominent. He was placed on mild diet, and one or two gentle aperients administered. His health remained undisturbed, and his spirits good up to the 19th of July, when, assisted by my colleague, Dr. Annan, and in the presence of several medical friends, the following operation was performed.

An enema having been previously administered, and gutt. lx. Tr. opii, given an hour previously, the patient was placed upon upon a suitable table, his pelvis inclining towards the left side, and an incision made through the skin and common integuments of three inches and a half in length, extending from three-fourths of an inch to the inside, and on a level with the antero-superior spinous process of the ilium, with a curve presenting downwards to about three-fourths of an inch above the external ring—a small vessel running to the skin was divided in the inner angle of this wound and secured. The glistening fibres of the fascia of the external oblique were then divided, according to a suggestion in the last edition of Dr. N. R. Smith's work on the arteries, in the same direction, but a half inch above Poupart's ligament. I now attempted to pass my finger under the edge of the external oblique and fascia transversalis, but found the border of the fascia of the external oblique presented such obstruction to free manipulation, by its resistance, that, to facilitate my farther progress, I was obliged to divide it with a buttoned bistoury, thus making a T incision in this fascia. The finger now passed readily down on the outer side of the internal ring, avoiding the cord, and after a little dissection with the nail, came in contact with the artery. Fearing, however, the soundness of the coats of the vessel thus low down, the sac of the peritoneum was gently pushed upwards and separated from the fascia transversalis for an inch and a half; and this membrane, together with the fibres of the internal oblique and transversalis divided by a buttoned bistoury on the finger for three-fourths of an inch. This sufficiently enlarged the wound for easy manipulation. The finger sought the artery about three inches from Poupart's ligament, and with one of Prof. Gibson's needles, armed with a ligature of two strands of sadler's silk, waxed but not twisted, an attempt was made to pass it from without inwards; finding the curve of the needle so great that I could not depress the handle sufficiently on the abdomen, I cautiously separated with the nail the vein from the artery, and passed it from within outwards. The ligature was then drawn tight with some difficulty at this depth, my assistant pressing on the knot until the second was tied. The pulsation immediately ceased in the tumor. The wound was brought together by two points of suture and a few adhesive straps. The operation lasted 25 minutes, and was borne extremely well by the patient. The patient, after the wound was dressed, was carried into a well-ventilated room, and placed on a bed with the limb of the side operated upon well flexed on the pelvis.

Complains only of fatigue and numbness of the right limb, with dull pain of the loins and hypogastric region—pulse 80—natural—at 7, P. M. Temperature of right foot, as tested by the thermometer, seven degrees lower than that of the opposite side—gave Tr. opii, gutt. xxx.

July 20th. Patient rested tolerably during the night—slept nearly four hours; countenance cheerful; some nausea and anorexia. Pulse 80, as yesterday; skin pleasant; complains of dull pain in the loins, and some cramps in the leg. Tumor decreased one-third in size; hard on its external side; slight pulsation at its inner and upper portion. Right leg florid, only two degrees lower in temperature than the left; ordered venesec. 14 oz. ℞. Morphiæ acet. gr. ij.; aquæ camphor. ℥iv.—dessert-spoonful every two hours. Towards evening his pulse rose to 114. Takes barley water made cold with ice.

21st. Appearance much altered; countenance anxious, haggard; eyes sunken and suffused; voice weak; nausea, constant retching; complete anorexia. Tongue reddish along the edges; thirst; skin bathed in perspiration; pulse 140, small; abdomen tympanitic; complains of sharp pain along the recti muscles, in the iliac region and about the loins; bladder irritable, trying to make water every ten minutes; venesec. oz. 14. ℞. Calomel grs. xv., pulv. opii, grs. ij. Added two drops of hydrocyanic acid to each dose of the solution of morphine; forty good leeches applied to the abdomen. At 7 P. M. the leech-bites were still running freely, and had afforded great relief; pulse 130; blood drawn this morning well buffed; still restless. Continue anodyne. Tumor as yesterday. Temperature equal on both sides.

22d. Countenance much improved; pain and uneasiness much less; abdomen still tympanitic; no movement of the bowels. Pulse 125, fuller but soft; tongue moist; thirst less, and asks for food. Still complains of pain in the back and abdomen; bladder still irritable; venesec. 8. oz. ℞. Cal. and opium as yesterday: hop fomentation over abdomen. 7. P. M. Still improving; skin pleasant; demands food; no passage; blood drawn in the morning lightly buffed; the bleeding made a sensible impression upon his pulse. ℞. Cal. grs. viij.; opii, gr. j.; chicken water, and milk iced. Continue solution.

23d. Passed a good night, and makes no complaint this morning excepting of his bladder; abdomen much softer; skin pleasant. Pulse 96, soft; tongue clean; no stool; little thirst; wound gives no pain; an erythematous blush surrounding it for some inches; complains of the limb. ℞. Sal Rochelle,

℥j.; aqua, ℥viij.: wine-glassful every hour. 7 P. M. Bowels not moved; complains of flatus. Ordered a simple enema.

24th. Looks as well as before the operation; makes no complaint; pulse 90, soft; tongue clean. Peritonitis fairly subdued, and we looked forward to a happy result; waking from a sleep at one o'clock he turned over in bed; his nurse a few minutes after was struck by his respiration and altered facies, and a quantity of blood in the bed. I saw him a few moments after, pale, restless, pulse scarcely perceptible, almost unconscious, his bed floating in blood—between two and three quarts. The hemorrhage had ceased; a compress was laid over it; nor did it return; he sunk rapidly, and died about 7 P. M. It was not deemed advisable to make any effort to secure the vessel, as the slightest shock would have determined dissolution under our hands.

*Autopsy eighteen hours after death.* Weather very warm. Head and face purplish; features swollen; bloody serum exuding from eyes; slight greenness about the clavicles and abdomen; rigidity considerable; muscles large and firm. The lips of the wound were united, excepting for a half inch on either side of the ligature; this space occupied by a clot of blood; the points of suture had held firmly, and there was no disturbance of parts.

The abdomen was opened by incisions from the umbilicus to the pubis, and to the edge of the ribs. Their flaps turned down exposed to the peritoneum. There was no effusion of serum; no adhesions; but over the tract of the artery, extending upwards towards the kidneys, and down into the pelvis to where the peritoneum invests the bladder, this membrane, in a space equal to the size of the hand, was of a bluish livid tint, more wrinkled than elsewhere, as if lightly parboiled, presenting numerous whitish yellow points, which could be scraped off with the nail; some flakes as large as a half-dime. The membrane was firm; no adhesion with the intestine. The peritonitis had been local, and was entirely subdued. The adhesions of the lips of the wound were then torn open; the skin and cellular tissue were the only parts adherent. The blood confined in the wound had insinuated itself upwards for some distance between the fascia transversalis and the peritoneum, as well as between this membrane and the fascia iliaca; about four ounces of clotted blood were removed from the wound. The artery was then exposed, and found with the ligature around it; when pressed upon, blood came from an opening immediately under the knot. It was then carefully dissected out and removed, from Poupart's ligament to the bifurcation of the aorta, and laid open, as well as the external iliac of the opposite side. They were equal in size; at least one-third of an inch in diameter; the inner coat of a creamy yellow hue; a little rough. The inner coat, where the ligature had been applied, on the posterior half of the artery, was wrinkled and crimped, so as to be smoothed out with some difficulty, but was not cut through, nor was there any effusion of lymph. On the anterior part was the opening, at least one-half the circumference of the vessel. The edges a little ragged, of a purplish hue, within a quarter of an inch of the opening; friable, and breaking easily under the nail. The outer coat was livid to the same extent; softened and more friable than the rest of the vessel; a soft clot below, but none above the ligature. The vein perfectly sound; no further separated from the artery than by the needle in passing the ligature; this was applied about an inch and a half below the bifurcation of the common iliac, and about the same distance from the origin of the epigastric. The tumor in the groin was next laid open and cleansed of its clots. It was about three inches in each diameter, the anterior wall formed by the fascia lata, its lateral and posterior walls by the muscles of the thigh; irregularly anfractuous. The profunda, which was given off very high up, immediately under Poupart's ligament, formed a portion of its posterior boundary. The artery, for an inch above where the tumor commenced, was filled with osseous concretions, grating under the scalpel, and pricking the fingers. The continuation of the superficial femoral where it emerged from the tumor, from the number and regularity of the circular atheromatous bands, looked more like the trachea than an artery, The aneurismal tumor was very thin and prominent towards the upper and inner part of the thigh. It contained between four and six ounces of soft black clotted blood, and as much firm fibrin arranged in layers penetrating into the anfractuosities of the tumor.

The arterial system was everywhere well developed; these vessels having more than their usual calibre. The heart was large and flabby, its walls thin, a little fatty; the valves all sound; aorta not dilated; other viscera of the chest and abdomen healthy. Head not examined.—*Maryland Med. and Surg. Journal.* September, 1842.

